# Forward Genetic Analysis of the Apicomplexan Cell Division Cycle in Toxoplasma gondii

**DOI:** 10.1371/journal.ppat.0040036

**Published:** 2008-02-15

**Authors:** Marc-Jan Gubbels, Margaret Lehmann, Mani Muthalagi, Maria E Jerome, Carrie F Brooks, Tomasz Szatanek, Jayme Flynn, Ben Parrot, Josh Radke, Boris Striepen, Michael W White

**Affiliations:** 1 Center for Tropical and Emerging Global Diseases and Department of Cellular Biology, University of Georgia, Athens, Georgia, United States of America; 2 Department of Biology, Boston College, Chestnut Hill, Massachusetts, United States of America; 3 Department of Veterinary Molecular Biology, Montana State University, Bozeman, Montana, United States of America; Albert Einstein College of Medicine, United States of America

## Abstract

Apicomplexa are obligate intracellular pathogens that have fine-tuned their proliferative strategies to match a large variety of host cells. A critical aspect of this adaptation is a flexible cell cycle that remains poorly understood at the mechanistic level. Here we describe a forward genetic dissection of the apicomplexan cell cycle using the *Toxoplasma* model. By high-throughput screening, we have isolated 165 temperature sensitive parasite growth mutants. Phenotypic analysis of these mutants suggests regulated progression through the parasite cell cycle with defined phases and checkpoints. These analyses also highlight the critical importance of the peculiar intranuclear spindle as the physical hub of cell cycle regulation. To link these phenotypes to parasite genes, we have developed a robust complementation system based on a genomic cosmid library. Using this approach, we have so far complemented 22 temperature sensitive mutants and identified 18 candidate loci, eight of which were independently confirmed using a set of sequenced and arrayed cosmids. For three of these loci we have identified the mutant allele. The genes identified include regulators of spindle formation, nuclear trafficking, and protein degradation. The genetic approach described here should be widely applicable to numerous essential aspects of parasite biology.

## Author Summary

Parasites of the phylum Apicomplexa cause numerous important diseases, including malaria, toxoplasmosis, and cryptosporidiosis. The ability to modify the genome of these parasites by transfection has been the technological key to unlock the biology of parasitic diseases at a molecular level. In this study we further extend the experimental possibilities for the study of apicomplexans by adapting a classic forward genetic approach for Toxoplasma gondii. We have developed protocols and reagents to generate large numbers of mutant parasites, screens to hone in on a subset of mutants of particular interest, and tools to identify the mutated genes that are responsible for the phenotype. Using this new approach, we have genetically dissected the way the parasite divides and multiplies within its host cell. This effort has yielded a series of highly informative mutants along the progression of the apicomplexan cell cycle and more than 20 genes involved in orchestrating parasite cell division. Importantly, this approach should allow unbiased genetic analysis of any part of parasite biology for which a screen can be devised using the *Toxoplasma* model.

## Introduction

Apicomplexans are highly successful protozoan parasites infecting a tremendous variety of vertebrate and invertebrate animals. In humans they are responsible for several important diseases, including malaria, toxoplasmosis, and cryptosporidiosis. A key to their success is their adaptation to a unique intracellular niche, which allows them ready access to nutrients while sheltering them from the immune system. Asexual parasite replication is restricted to this intracellular part of the life cycle, and intermediate stages of intracellular replication in many species lack the machinery to infect new host cells. It is therefore critical for the parasite to time its cell division and the formation of invasive forms to coincide precisely with host cell egress. To adapt to a variety of specific host cell niches, apicomplexa have developed several specialized cell division modes. These division modes are based on a ‘flexible' cell and division cycle program that can actively coordinate DNA synthesis and chromosome segregation, while at the same time suspending nuclear division and/or cytokinesis until the last step of the replication cycle (see [1,2] for recent detailed reviews of apicomplexan cell division and cell cycle control). Thus, re-initiation(s) of DNA synthesis prior to the completion of cytokinesis occurs naturally in these parasites. The molecular mechanisms that count the rounds of DNA synthesis and provide the proper timing of parasite budding remain one of the compelling mysteries of these parasites. Database mining for factors commonly associated with eukaryotic cell cycle control has identified an extensive set of candidate regulatory proteins in apicomplexan parasites (*e.g.* cyclins, CDKs, MAPKs [3–6]). While these findings predict that cell cycle checkpoints exist in Apicomplexa, they do not provide information about where checkpoints function or how these controls operate to safeguard the diverse strategies utilized by these parasites.

Here we describe the genetic analysis of the apicomplexan cell division machinery in Toxoplasma gondii. While the simple binary division of *Toxoplasma* tachyzoites (also termed endodyogeny) offers an attractive model system, we expect these studies to apply broadly to the replication of other pathogens in this phylum, such as *Plasmodium*, *Eimeria*, and *Cryptosporidium*, where our knowledge of the parasite cell cycle is equally deficient. Using chemical mutagenesis and a high-throughput replica assay, we have isolated a large collection of temperature sensitive (*ts*) parasite mutants. Our phenotypic analyses map these mutants to specific steps of the parasite cell and division cycle. To identify the underlying genes we have developed a robust complementation model employing cosmid transformation. This approach allowed us to link mutant phenotypes to specific point mutations in parasite genes.

## Results

### Production and Isolation of a Large Collection of Conditional Growth Mutants

**Figure 1:**
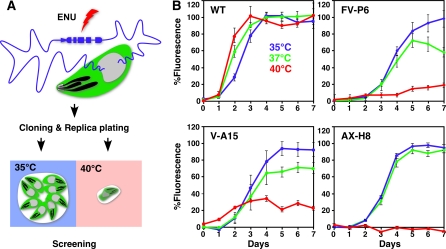
Isolation of *T. gondii ts* Mutants (A) A fluorescent reporter strain of T. gondii is mutagenized by exposure to ENU and immediately cloned in 384 (or 96)-well plates. Plates are replicated and scored for growth at the permissive (35°C) and restrictive (40°C) temperature by measuring fluorescence (or by visual inspection). (B) Fluorescence growth curves at 35 (blue), 37 (green), and 40°C (red) are shown for wild type and three representative *ts* mutants.

A pool of conditional growth mutants was established by chemical mutagenesis using *N*-nitroso-*N*-ethylurea (ENU), an agent used successfully in the past to generate point mutations in the T. gondii genome [7–10]. ENU was applied at a dose inducing 60–70% parasite killing (measured through plaque assay; results not shown). We estimate this dose to induce 10–100 mutations per genome based on the incidence of mutations in hypoxanthine-xanthine-guanosine phosphoribosyltransferase (HXGPRT [11,12]), which we measured by following the emergence of resistance to the HXGPRT activated prodrug 6-thioxanthine (10^−5^ in our experiments, also see [7]). To enable high-throughput screening we developed a well plate replica assay (see Figure 1 for a schematic outline of the strategy). Following mutagenesis, parasites were immediately cloned into 384 (or 96) well plates seeded with HFF cells to avoid competition with wild type parasites and allowed to expand at the permissive temperature (34°C or 35°C). To identify mutants, plates were duplicated using a pin-tool transferring 5 (or 20) μl parasite suspension into two new plates. One plate was kept at the permissive temperature, whereas the replicate was placed at 40°C (restrictive temperature). Two approaches were applied to detect temperature sensitivity: visual microscopic inspection of wells (mutagenizing parent strain RH/*hxgprt*−) and measurement of fluorescence (mutagenizing the autofluorescent reporter strain 2F-1-YFP2 [13]). Fluorescence was measured after 4 days (40°C) and 7 days (35°C). The values were normalized against the 2F-1-YFP2-parent line included in each plate, and corresponding wells were compared for differential growth at permissive and restrictive temperatures using an automated script (a fluorescence increase below 20% was scored as a growth phenotype). Parasites from wells exhibiting growth at 35°C but not at 40°C were expanded, and temperature sensitivity was re-confirmed (see Figure 1B for examples; mutants with an identifier that starts with a letter were obtained through the fluorescence screen, those that start with a number through the visual screen).

In total we have identified 165 *ts* mutants from ∼60,000 clones produced (see Table S1). Approximately 5% of the primary clonal isolates selected in the screens were confirmed by secondary analysis to show conditional growth arrest at the restrictive temperature (165/2960). False positives arose equally using either detection method and were due to the liberal selection for potential growth mutants and also as the result of replica-pin transfer failures (∼5%). The overall frequency of confirmed *ts* mutants generated by our combined screens is lower (0.26%) than the value obtained in an earlier pilot screen (1.1%, [14]), which was much smaller in scale (∼3,600 total ENU clones screened) and employed less stringent criteria for validating conditional growth. The *ts* mutants produced here display a lower reversion to wild type growth than our earlier study with >85% of clones in the current screen having reversion frequencies that are <10^−6^ and many isolates revert at <10^−7^ (see Table S2). The *ts* mutants generated by this and our earlier study [14] display almost exclusively conditional-lethality. However, a few examples of mutants where viability is retained following temperature shift were observed; as noted in other eukaryotic models [15,16] these are mostly G1 mutants (see below).

### Phenotypic Analysis of Cell Cycle Mutants

**Figure 2:**
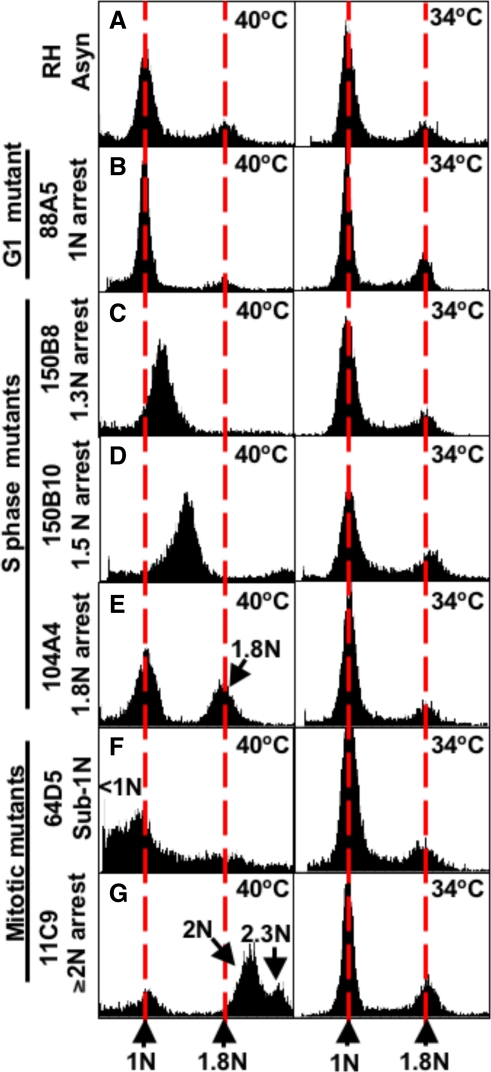
Distribution of Parasite Genomic DNA Content at Permissive and Restricted Temperatures for Selected *ts* Mutants Mutant clones were grown at 34°C and 40°C for various times (16–36 h, based on individual phenotype) prior to harvest and ethanol fixation. DNA was stained with SYTOX Green dye and measured using a FACS calibur (BD). The cytometer was set to mode fluorescence and calibrated to the 1N population of asynchronous RH wild type parasites; red dashed lines reference 1N and 1.8N fluorescence peaks in the asynchronous controls. DNA fluorescence was measured in FL-1 linear scale (*x*-axis) and 10,000 events were collected for each histogram. In comparison to asynchronous controls (A), a selection of *ts* mutants (grown at 34°C and 40°C) that cell cycle arrest with different genomic contents are presented: (B) mutant 88A5 arrests with a predominant 1N DNA content (G1 phase mutant), mutants 150B8, 150B10, and 104A4 show altered DNA contents that are intermediate with respect to normal haploid or diploid genomic contents (C, D, and E), mis-segregation mutant 64D5 shows significant chromosome loss ([F], <1N), while mitotic mutant 11C9 arrests with a predominant diploid DNA content (G).

**Figure 3:**
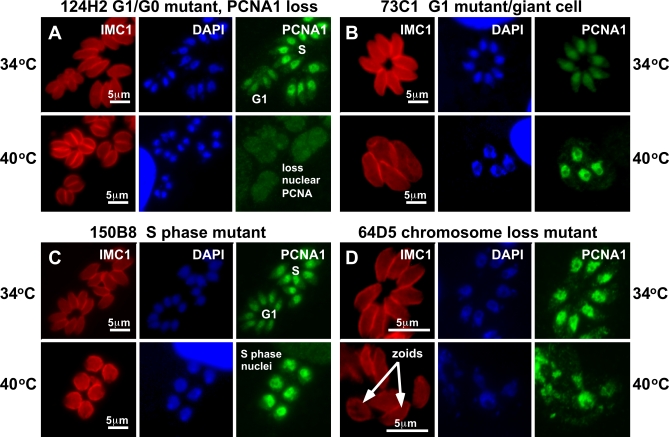
Representative Examples of G1, S, and Mis-Segregation Mutants At various times post-infection at 34°C and 40°C (16–36 h, chosen based on phenotypic analysis of each mutant), *ts* mutants fixed and co-stained with antibodies αIMC1 (red, inner membrane complex and daughter buds) and αPCNA1 (green, replication foci) and also with DAPI (blue, DNA). (A and B) Mutants 124GH2 and 73C1 arrest at 40°C after several divisions with a predominant 1N DNA content based on FACS analysis (not shown), indicating synchronous arrest in the G1 or G0 phase. Growth arrested mutant 124GH2 parasites had a normal shape and size and a single nucleus; however, in >75% of the vacuoles, parasites showed an unusual loss of nuclear PCNA1 staining, suggesting that theses parasite could be arresting in a “G0” state. The 34°C images shown for this mutant capture the normal range of nuclear staining observed for native TgPCNA1 in asynchronously growing populations. By contrast, parasite size and shape were severely altered when mutant 73C1 was shifted to the restrictive temperature. (C) By FACS analysis (Figure 2C), mutant 150B parasites that were arrested at 40°C possessed a partially duplicated genome, and this finding was consistent with the large centrally located nucleus that stained intensely with αPCNA antibody. These phenotypic features are consistent with growth arrest in S phase. (D) Mutant 64D5 is representative of five chromosome mis-segregation mutants that all show a prominent sub-1N DNA distribution by FACS analysis (Figure 2F) and by IFA analysis show fragmentation of nuclear material in growth arrested parasites. At a low level, parasites containing little or no DAPI staining material were also observed in these populations (zoids, arrows).

**Figure 4:**
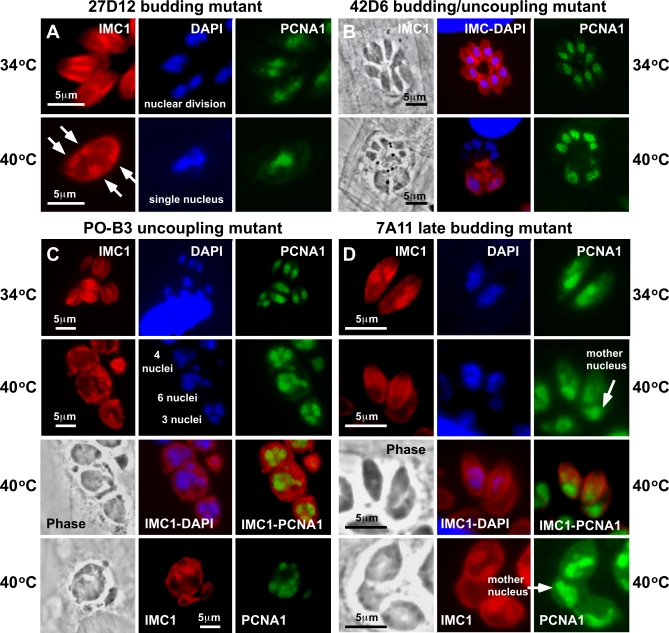
Representative Examples of Mutants with Defects in Karyokinesis and Parasite Budding Several mutants characterized in our screens display defects in daughter budding when shifted to the restrictive temperature. (A) Mutant 27D12 shows multiple small IMC1 staining bodies (often closely associated with the plasmalemma, arrows), indicating abortive budding at an early stage. When arrested at 40°C, these normal-sized and shaped parasites had single or duplicated nuclei (but never formed syncytial cells containing multiple nuclei). In time-matched 34°C controls, parasites were able to complete two to three divisions. (B, C, and D) Errors in karyokinesis and parasite budding were detected in several mutants, which led to an uncoupling of these processes in some cases. Mutants 42D6 and PO-B3 show multiple abnormal budding structures and show progressive nuclear reduplication leading to syncytial cells with many nuclei (cells with variable nuclei numbers are indicated in PO-B3 image). Mutant 7A11 forms more complete daughter buds that are associated with at least one round of nuclear division. Premature budding before completion of the previous cytokinesis is observed in many vacuoles, which causes retention of a mother cell and nuclear structure (arrow). Two independent example vacuoles are shown for mutants 7A11 and PO-B3. See Figure 3 for details on antibodies used.

Mutants were analyzed for uniform population changes in DNA content measured by flow cytometry (FACS). Phenotypes were further characterized by immunofluorescence assays (IFAs) identifying distinctive cellular and nuclear morphologies that developed at the restrictive temperature (see Table S2 for detailed individual descriptions of all mutants examined). Specific antibody reagents used in IFA analyses were directed against centrin as a marker for the number and position of centrosomes [17–19], membrane occupation recognition nexus (MORN)1, a marker for spindle morphology and budding [19,20], TgPCNA1, an essential element of the nuclear DNA replication complex [21,22], and inner membrane complex (IMC)1 and 3, components of the membrane skeleton that served as a budding marker [23–25]. The majority of parasite mutants examined had phenotypes characteristic of growth arrest in a specific cell cycle phase (*i.e.* >75% of the parasite population examined by FACS or IFA show a similar phenotype, see Figures 2, 3, and 4 for representative examples). In comparison to similar efforts in *Saccharomyces* [26,27], fewer general growth mutants were produced by our screens (the overall yield of *ts* mutants is also lower). However, several non-cell cycle mutants were identified including *e.g.* F-P2, a mutant with normal intracellular development but a severe invasion and egress defect (M. J. Gubbels and B. Striepen, unpublished data).

Based on their shared terminal phenotypes, mutants were readily classified into groups. These groups express defects in mechanisms active across the full spectrum of events in parasite replication. As expected, cell cycle mutants were isolated that possess a dominant haploid DNA content (1N) at the restricted temperature. The Sytox Green-FACS histograms for mutant 88A5 (Figure 2B, 34°C versus 40°C) are representative of this large group of G1 phase mutants. In addition to a 1N DNA content, G1 mutants possessed a single nucleus and had no or few internal daughter forms at the time of growth arrest (data not shown, see Table S2). Outside this core set of phenotypic features, G1 mutants in our collection express diverse secondary characteristics that include alterations in terminal cell size (*e.g.* mutant 73C1, Figure 3B), differences in the number of cell divisions before growth arrest, and variations in intermediate temperature sensitivity. The G1 class is the only group where non-lethal *ts* mutants were isolated in our screen. Mutants 63H4 and 31F1 stop within a single cell division in the G1 phase when shifted to 40°C and can be held at this temperature for 24 hours without significant loss of viability (116% or 92% plaques formed compared to controls maintained constantly at the permissive temperature. Note that most mutants show poor recovery in this assay *e.g.* 88A5 or 87A10 with 1 or 4%, respectively). Two unusual mutants classified in the G1 group display complete loss of nuclear proliferating cell nuclear antigen (PCNA)1 staining at the restrictive temperature (*e.g* mutant 124H2, Figure 3A).

A second group of *ts* mutants in the collection arrest upon shift to 40°C with an intermediate DNA content (>1N but <2N by Sytox Green-FACS) consistent with S phase arrest. Five *ts* mutants representing this class (*e.g.* 150B8, Figure 2C) arrest with a 30% increase over the haploid DNA content at the restrictive temperature; this phenotype is very similar to RH^TK+^-parasites blocked by thymidine treatment in early S [28]. Arrested cells had large, centrally located nuclei and the DAPI and nuclear PCNA1 staining patterns observed (Figure 3C) were consistent with the S phase assignment [22]. Two additional S phase mutants deserve mention here: mutant 150B10 possessed a mid-S phase DNA content at the time of growth arrest (Figure 2D), while mutant 104A4 (Figure 2E) showed a unique bimodal distribution of parasites into equal 1N and 1.8N subpopulations.

Internal budding is a unique feature of apicomplexan replication, and >30% of the initial *ts* mutants characterized by IFA and FACS display defects in cytokinesis, some of these show simultaneous defects in karyokinesis. Mutant 64D5 is representative of five mutants that have defects in chromosome segregation. A subpopulation with a sub-1N DNA content by FACS (Figure 2F) as well as microscopic evidence for zoid formation (anucleate daughter cell [29], Figure 3D) are key characteristics of these mis-segregation mutants. A second group of M-phase mutants shows defects in the early stages of budding. Multiple small IMC1 staining structures formed but failed to develop fully when the parasites were shifted to the restrictive temperature (Figure 4A). Other budding mutants developed defects that interfered with the resolution of the mature daughters from the mother cell. Apparently this did not prevent a second round of cell division from unfolding suggesting cell cycle counting mechanisms were still active in this late budding mutant (bud-within-bud mutant 7A11, Figure 4D). The early and late budding mutant examples shown here maintained normal numbers of nuclei per parasite (one or two), whereas in other mutants isolated by our screens a catastrophic breakdown of the coordination between cytokinesis and karyokinesis occurred at the restrictive temperature. The three examples of this type of mutant shown here were promiscuous for chromosome re-initiation, which led to the formation of multiple or very large nuclei (42D6 and PO-B3, Figure 4B and 4C, and V-A15 see below). In each mutant, the mitotic spindle apparatus appears disorganized based on MORN1 antibody staining (all three mutants had similar staining as shown for mutant V-A15 below). Interestingly, similar abnormal budding and massive DNA over-replication has been observed in parasites where the spindle has been disrupted pharmacologically [29], suggesting that spindle defects might be a key feature of the uncoupling phenotype expressed by these *ts* mutants.

### A *Toxoplasma* Cosmid Library for Phenotypic Complementation

To identify the genes underlying the mutant phenotypes, we employed phenotypic complementation using a wild-type genomic DNA library. Several T. gondii libraries have been generated for this purpose and some success has been reported [25,30–32]. However, in our initial experiments we found that the established cDNA library strategies were not sufficiently robust to provide complementation on a regular basis with the *ts* mutant pool produced in this study. This is likely due to limitations of cDNA based plasmid libraries with respect to pool redundancy and insert size (many cell cycle factors are encoded by low abundant mRNAs). To establish a complementation model that is independent of gene size and differential transcript levels, we generated a RH genomic DNA cosmid library.

**Figure 5:**
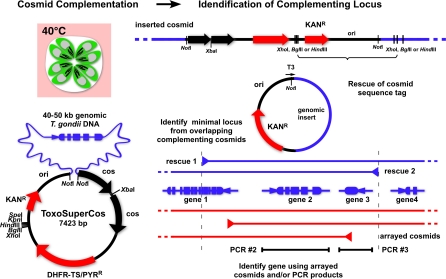
Schematic Outline of the Cosmid Complementation Strategy Confirmed mutants are transfected with the ToxoSuperCos cosmid library (containing 40–50 kb inserts of WT RH genomic DNA), and selected under pyrimethamine at the restrictive temperature. Sequence tags of complementing cosmids are rescued into plasmid from parasite genomic DNA by restriction, dilute ligation and selection for kanamycin resistance after electroporation into E. coli (insert can be sequenced using T3 primer). Querying ToxoDB using independent sequence tags (shown in blue) identifies a minimal complementing locus (dashed vertical lines). The locus is confirmed and narrowed using a library of arrayed and sequenced cosmids (shown in red) and/or PCR products (shown in black) covering individual genes.

T. gondii does not maintain stable episomes precluding a simple shuttle of cosmids between parasite and E. coli to isolate complementing sequences. We therefore adapted a strategy based on cosmid insertion and rescue of a sequence tag (see Figure 5 for a schematic outline). Our library was constructed in ToxoSuperCos, a double cos-site plasmid based on the commercial SuperCos1 construct (Stratagene). However, several features were engineered into ToxoSuperCos to facilitate rescue after insertion. The T. gondii pyrimethamine resistance marker DHFR-TSm2m3 [33] was included to select for stable cosmid integration into the parasite genome preserving the backbone. This is important as plasmid rescue requires a bacterial origin of replication and a drug resistance marker for selection in bacteria ([34], see Figure 5). We chose a kanamycin resistance gene, as most parasites used for screening already contain plasmid DNA harboring ampicillin resistance genes (due to previous genetic engineering to introduce reporters and markers). To further facilitate rescue, a polylinker was incorporated providing a broader choice of restriction sites for genomic excision of rescue tags.

The resulting library consists of 1.25x10^6^ independent cosmid clones providing ∼900-fold genomic coverage. Using pyrimethamine selection and parasite plaque assays, the transformation efficiency was determined to be 0.3% (which is comparable to plasmid based transfection taking the larger size of the cosmid construct into account). Using the protocol described in the materials and methods section, 12-fold coverage of the T. gondii genome should be achieved in each transfection experiment. To validate these calculations, we transfected the T. gondii RH-*hxgprt-* deletion mutant with the cosmid library and selected for complementation by treatment with mycophenolic acid [11] and pyrimethamine. Five out of five electroporations resulted in the isolation of viable and inheritably mycophenolic acid resistant parasites. Clonal lines were established from three independent transfections, and the wild type HXGPRT locus (absent from the mutant but present in the library) was detected by PCR and sequencing of the PCR product in ten out of fifteen clones (Figure S1). Further PCR-based analyses indicated that complementation occurs through heterologous insertion of an additional wild type copy rather than homologous gene replacement of the mutant locus (even in experiments where no selection for the pyrimethamine marker is applied).

### Complementation Analysis of the Mitotic Mutant V-A15

**Figure 6:**
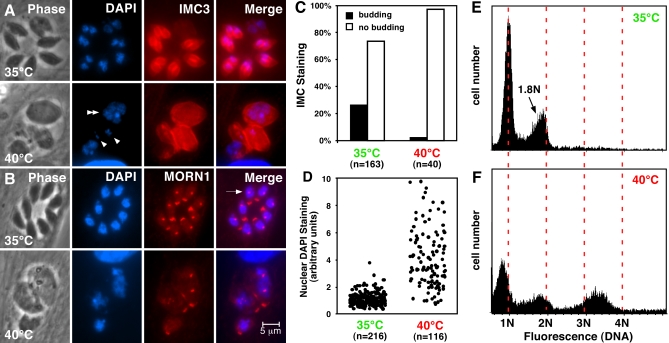
Mutant V-A15 Shows Temperature Sensitive Growth due to Severe Defects in Mitosis at the Restrictive Temperature (A) Immunofluorescence analysis of V-A15 using an anti-serum against the inner membrane complex marker IMC3 [25]: at the permissive temperature internal daughter buds (IMC3) are frequently observed, but fail to form at the restrictive temperature (36 h, quantification shown in [C]). Note that V-A15 forms very large cells at 40°C (Phase, all panels shown at same magnification) and that nuclear DNA (DAPI) is not or unequally segregated resulting in large (double arrowhead) and small nuclei or nuclear fragments (arrowheads). Nuclear size was quantified by image analysis and is shown in (D); note wider size distribution at the restrictive temperature. (B) Immunofluorescence analysis of V-A15 using an antibody to MORN1 [20]. At the permissive temperature a clearly defined centrocone can be identified in each nucleus (Merge, arrow), at the restrictive temperature this organization is lost. (E and F) DNA content was also quantified by flow cytometry (see Figure 2 for experimental details). Distinct populations with DNA contents of <1N and >2N arise at the restrictive temperature (F). Note that flow cytometry might not detect very large parasites due to a filtering step, and that image analysis (D) will likely underestimate the number of <1N cells.

Having established that the ToxoSuperCos library robustly complements the HXGPRT mutant, we then tested its ability to genetically rescue *ts* mutants. Here we describe experiments with mutant V-A15 in detail as an example, but note that subsequently numerous additional *ts* mutants have been successfully complemented with this library (see below). Mutant V-A15 shows tight temperature sensitivity, with a modest growth delay at 35°C, significant inhibition at 37°C and a severe defect at 40°C (Figure 1B; the reversion frequency of this mutant was measured by plaque assay to be <10^−7^). IFA and FACS analyses revealed that growth inhibition is due to severe mitotic defects. V-A15 parasites shifted to the restrictive temperature failed to complete mitosis which led to polyploid nuclei and/or chromosome loss (Figure 6B, 6D, and 6F; note that increase in nuclear size goes along with an increase in cell size). While internal daughter buds were readily observed in parasites grown at the permissive temperature by staining with IMC3 (∼25% of all vacuoles, Figure 6A and 6C), no clear IMC3 structures were formed at the restrictive temperature. Because features of this phenotype are consistent with a defect in the mitotic spindle, we analyzed mutants for the cellular distribution of MORN1, a marker of the nuclear spindle compartment (centrocone [20]). In parasites grown at the permissive temperature, the centrocone was clearly detected in each nucleus, while under temperature restrictive conditions MORN1 nuclear staining appeared disorganized or was entirely absent from some of the nuclei.

**Figure 7:**
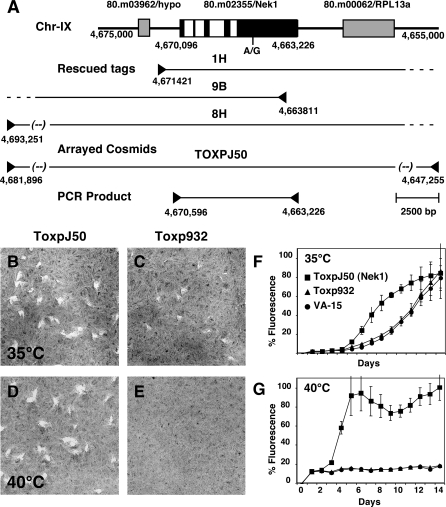
Cosmids Complementing Mutant V-A15 Map to the Locus of the T. gondii NimA Kinase Nek1 Mutant V-A15 was transfected with the ToxoSuperCos library and selected as described in Figure 5. (A) Sequence tags of complementing cosmids (from three independent transfections) were rescued and sequenced; they map to a locus on chromosome IX and show an overlapping region of 7,610 bp containing gene model 80.m002355 annotated as NimA related kinase. (B) Mutant V-A15 was transfected with cosmids ToxpJ50 (covering the identified locus on Chr. IX, [A]) or control cosmid Toxp930 (Chr. VIIb) and selected for pyrimethamine resistance at the permissive temperature. The resulting stable transgenic populations were tested for temperature sensitivity by plaque assay (B–E) and fluorescence assay (F and G). Note that ToxpJ50 complements while Toxp930 does not (as the mutant these transgenics show a slight growth defect even at the permissive temperature, resulting in smaller plaques [C] and slower increase in fluorescence [F]).

To identify the underlying genetic locus, mutant V-A15 was transfected with the ToxoSuperCos library in five independent electroporations, inoculated into confluent HFF cultures and allowed to recover for 24 hrs at 35°C. The flasks were subsequently transferred to 40°C to select for growth restoration (phenotypic complementation), and pyrimethamine was added to select for stable cosmid backbone integration. Stable temperature and drug resistant parasites emerged in four out of five flasks. To identify the complementing cosmid sequence, we used plasmid rescue of a sequence tag (see Figure 5 for an outline of this strategy). Genomic DNA of complemented parasite lines was extracted and digested with *Spe*I, *Hin*dIII or *Bgl*II, self-ligated overnight, and then electroporated into E. coli. Kanamycin resistant colonies were recovered for all four complementations using at least two out of the three enzymes used. Plasmid DNA was isolated and the genomic inserts were end-sequenced using the T3 primer. Three out of the four sequence tags (each from independent complementation experiments) mapped to a 30,000 bp locus on chromosome IX and a region of 6.7 kb could be identified that was shared between all three rescued tags (see Figure 7A; the fourth complement mapped to Chr VIII 1225114 bp minus strand, and was not studied further). The overlapping region contains a single predicted gene model (80.m02355) encoding a putative NIMA-related kinase with close similarity to Plasmodium falciparum Nek1 [6,35].

### Locus Confirmation and Identification of a Point Mutation in TgNek1

Rescue of a sequence tag as described above readily identifies a candidate complementing locus, yet only a small portion of the complementing sequence is cloned into plasmid, which is often not sufficient to independently confirm the result by re-complementation. However, the ToxoSuperCos library (along with a second library constructed by Dan Howe and David Sibley, pSCBle, http://toxomap.wustl.edu/) has been end-sequenced in the course of the T. gondii genome project, and the cosmid tiling (covering essentially the entire genome) can be viewed through ToxoDB (http://www.toxodb.org/ ancillary genome browser). To test if the locus identified is indeed sufficient to complement the mutation, V-A15 was transfected with cosmid ToxPJ50 (containing the TgNek1 locus, Figure 7A) or ToxP932 (an unrelated control cosmid; Chr VIIb 1,484,413bp-1,521,843bp). Stable transgenics were established by pyrimethamine selection at the permissive temperature. These parasites were scored for temperature sensitivity by plaque assay (parasite growth is indicated by host cell lysis resulting in clear plaques in the fibroblast monolayer) and fluorescence growth assay (measuring the fluorescence of the yellow fluorescent protein (YFP)-YFP transgene expressed by the parasites [13]). ToxPJ50 restores robust growth at 40°C, while the control transgenic is indistinguishable from the mutant (Figure 7B–7G, note that complementation with ToxPJ50 already confers a modest growth advantage at 35°C, panel F).

**Figure 8:**
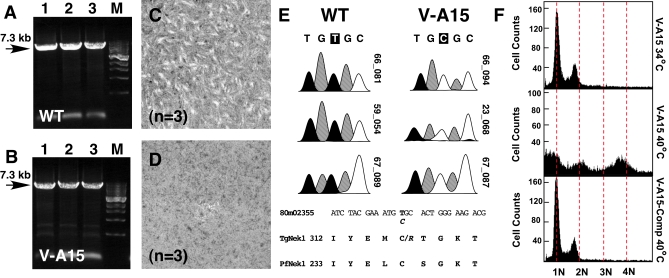
A Point Mutation in T. gondii Nek1 Is Responsible for the Temperature Sensitive Cell Division Defect in Mutant V-A15 The locus of TgNek1 was amplified from wild type (A) and V-A15 (B) genomic DNA in three independent reactions. V-A15 was transfected using these PCR products and incubated at the restrictive temperature and evaluated by plaque assay. WT PCR product rescued in three out of three independent transfections ([C], a single representative example shown here), while no plaques were observed using V-A15 PCR product (D). Both PCR products were sequenced on both strands. (E) A single nucleotide change was observed changing a cysteine to an arginine codon in a conserved segment of the Nek1 coding sequence (a limited alignment with the P. falciparum Nek1 gene is shown). (F) The DNA profile of mutant V-A15 and its complement (derived from the transfection described in (C)) was evaluated at the 34°C and 40°C. Note that complementation restores the wild type profile.

The complementation data indicate that the TgNek1 locus is the site of the mutation causing the V-A15 phenotype, or alternatively, that this locus can act as a suppressor. To distinguish these two possibilities, we amplified the locus by PCR from both wild-type parent and mutant V-A15, transfected the mutant with each allelic gene fragment and scored for complementation by plaque assay. PCR product from wild-type complemented in three out of three independent experiments, while no growth was observed with the V-A15 derived gene fragment (Figure 8A–8D), suggesting that the mutation is localized within this locus. Importantly, complementation with the wild type PCR fragment also restores the FACS DNA profile of parasites grown at 40°C to the typical wild type distribution (Figure 8F). The 7.2 kb PCR fragments were sequenced on both strands and compared to the genome sequence as well as each other. A single base pair change distinguishes the sequence of the mutant from RH wild-type. This change of a T to C lies within the Nek1 coding region and changes a cysteine to an arginine in a highly conserved portion of the predicted protein (Figure 8E shows the mutation along with a short alignment of Nek1 from T. gondii and P. falciparum). Taken together, these data indicate that a point mutation in TgNek1 is responsible for the severe mitotic defects observed in this mutant.

### Cosmid Complementation of a Variety of T. gondii Cell Division Mutants

**Figure 9:**
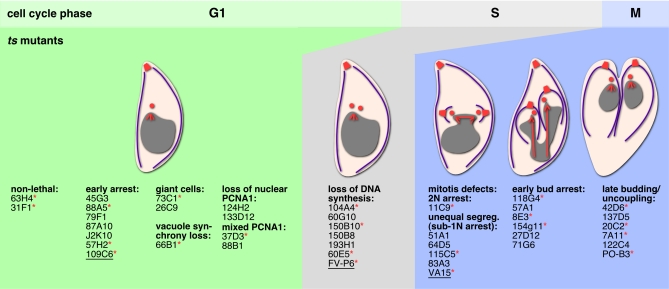
Summary of Phenotypic Classification of *ts* Cell Cycle Mutants A schematic outline of the tachyzoite cell cycle (60% G1, 30% S, and 10% M-phase) is shown at the top. Nuclei are shown in grey, microtubular structures in red (conoid, centrosome, and spindle; subpellicular microtubules were omitted for simplicity) and the inner membrane complex in purple. Mutants successfully complemented at this point in time are denoted by an asterisk.

Encouraged by our success in complementation analysis of mutant tsV-A15, we have broadened our efforts to ultimately identify the genes affected in all of the 165 *ts* mutants isolated in this screen. Figure 9 summarizes phenotypic and genetic analyses for the first group of 41 mutants (7 additional mutants showed excessive reversion frequencies and were excluded from further analysis). While these studies are still ongoing, thus far, we have observed complementation (indicated by asterisks) for 22 out of 24 attempted mutants, and a candidate locus has been identified by marker rescue for 18 mutants (see Table 1). For 8 mutants the locus has been independently confirmed by successful re-complementation using the respective arrayed cosmid, and for three genes the mutant allele has been identified by sequencing as described above for V-A15 (underlined in Figure 9; this data is summarized in detail in Table 1).

**Table 1:**
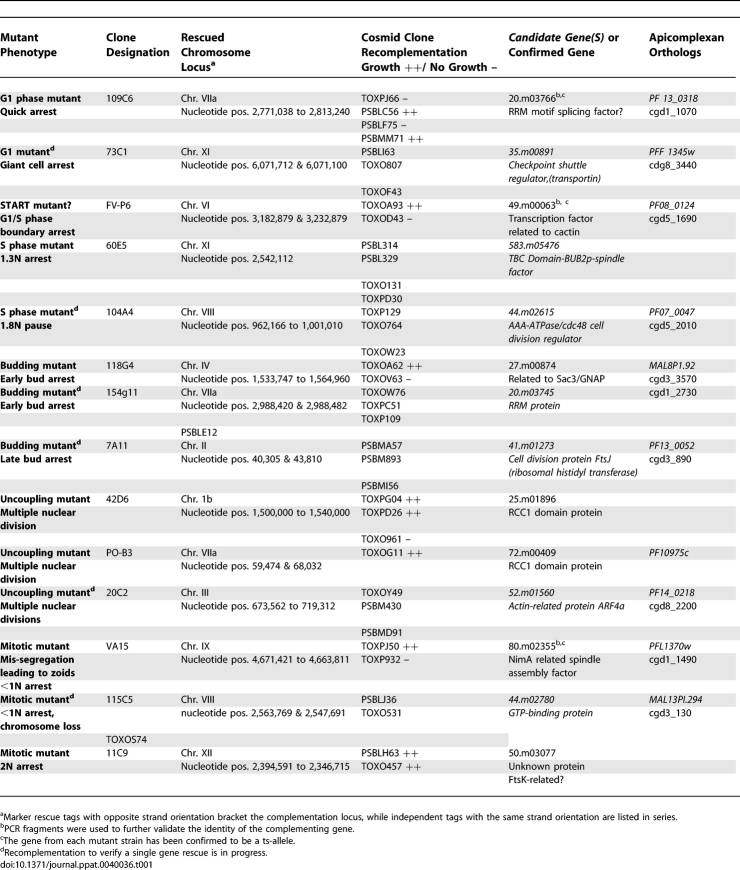
Summary of Cosmid Complementation for a Selection of Distinct *ts* Mutants

The genes identified through our mutant analyses encode proteins with potential functions in a wide array of parasite cell cycle mechanisms. Eleven of the 14 T. gondii genes summarized in Table 1 have clear homologs in other apicomplexan genomes as identified by OrthoMCL. In several cases, they represent orthologs of known cell cycle factors from other eukaryotes. Orthologs of the NimA-related kinase that complements V-A15 have well described roles in centrosome biology and mitotic entry [35–38]. Other examples include the *Toxoplasma* protein encoded by 27.m00873, which appears to belong to the Sac3/GNAP family. The SAC3 gene product in yeast is a nuclear factor that is required for mitotic progression. Defects in this gene cause errors in yeast budding and mitotic delay [39], which compares with the failure of mutant 118G4 to properly progress into mitosis and budding. An AAA-ATPase (44.m0215) is present in the locus complementing S phase mutant 104A4. AAA-ATPases fold and unfold proteins and often act as gatekeepers of protein degradation [40]. Mutations in AAA-ATPases (most notably cdc48) have been shown to result in mitotic arrest [41]. A homolog of the nuclear actin ARP4a is present in the rescue locus of mitotic uncoupling mutant 20C2. Nuclear actins are involved in transcriptional control and DNA repair and are required for stable attachment of the kinetochore to the mitotic chromosome [42]. Mutations in ARP4a cause defects in the intranuclear spindle and lead to an arrest of the yeast cell cycle in the G2 and mitotic phases.

A second set of genes encodes products that harbor protein domains often found in regulatory proteins but which otherwise appear to be unique to Apicomplexa. Gene 583.m05476 features a TBC domain, an activator domain known to regulate rab-like GTPases and is a motif also found in the yeast spindle factor BUB2p [43]. Two mutants, 42D6 and PO-B3, were complemented by proteins harboring RCC1-domains (regulator of chromosome condensation, 25.m01896 and 72.m00409). RCC1 proteins control nuclear transport and mitotic progression through nucleotide exchange of ran-GTPases [44]. Interestingly, another unrelated T. gondii RCC1 protein was recently identified as a non-essential protein that when mutated attenuates the virulence of Type I strains in the mouse model [44].

Gene 20.m03766, which complements the G1 mutant 109C6, contains an RNA recognition motif (RRM) present in RNA splicing factors, among others. Key features of the mutant 109C6 phenotype share similarities to the defects observed in yeast splicing mutants [45], and epitope tagged TgRRM-protein exclusively localizes to the tachyzoite nucleus (not shown) consistent with a putative role in pre-mRNA splicing. Sequencing of the RRM-protein allele from mutant 109C6 reveals a single base change that alters a highly conserved tyrosine residue in the RRM motif (169-Y to N), indicating that this mutation is likely responsible for the G1 arrest. In support of this hypothesis, we were unable to complement mutant 109C6 with a gene fragment carrying the mutant 20.m03766 allele.

## Discussion

Mutant analysis and genetic complementation are powerful strategies to link specific genes to biological pathways [46–48]. Efforts to develop these methods in *Toxoplasma* have resulted in an episome-based protocol [49] and an insertion based approach [31]. In the latter, phage recombination [50] was used to mobilize cDNA fragments integrated into parasite transformants [30–32]. While these earlier methods achieved some success, the redundancy inherent to cDNA libraries limited the complementation success (B. Striepen and M. White, unpublished data). The genomic-DNA based approach introduced here benefits from the large inserts carried by cosmid vectors to deliver genes in their natural chromosome organization. As a consequence of these improvements, our success rate for genetic rescue of *ts* growth mutants has improved dramatically. Here we report on the complementation of a diverse selection of mutants (>20 mutants, with a failure rate <5%). The genes identified in these experiments represent a wide range of coding and genomic sizes. Consistent with a mostly regulatory role of their products, the level of transcript for these genes is generally modest based on microarray analysis of tachyzoite gene expression (see summary in Figure S2).

One of the mutants complemented in this study (11C9) was the subject of an earlier cDNA-based complementation experiment that yielded a suppressor (TgXPMC2) of the genetic defect in this mutant [32]. Multiple complementation experiments of 11C9 using the cosmid library have repeatedly identified a single *Toxoplasma* gene, 50.m03077 (Table 1), which was likely underrepresented in the previously used cDNA libraries due to its large size and low expression level (<100 units average fluorescence intensity in tachyzoite microarrays for 50.m03077 versus ∼15,000 units for GRA-1, which was the promoter source used to drive expression in the cDNA libraries [31,32]). Likewise, we have not re-isolated TgXPMC2 by cosmid complementation. Because gene expression from cosmids relies on native regulatory regions, we believe there is a higher likelihood that the genes identified through this approach will represent the defective gene rather than a suppressor. In the three *ts* mutants (109C6, VA-15, and FV-P6) where this question was examined so far sequencing and functional testing of the corresponding mutant alleles confirms this prediction. In summary, the cosmid system provides robust complementation for a broad range of genes. Furthermore, the identified loci can be readily biologically validated taking advantage of an extensive set of end-sequenced and tiled cosmids that provide essentially full genome coverage. The protocols and reagents developed in the course of this study should allow future forward genetic analysis of any essential aspect of parasite biology for which a mutant screen can be devised.

Tachyzoite growth rates differ dramatically among parasite strains and growth rate is a key virulence determinant in *Toxoplasma* [22,51]. Despite the obvious importance of growth control, how the parasite regulates growth and cell division remains largely unknown. A series of defined biochemical controls and checkpoints regulating progression through one cell cycle phase to the next have been established for a variety of eukaryotic models [52–54]. The catastrophic break down of cell cycle coordination observed in T. gondii in the course of certain drug treatments has lead to the hypothesis that there might be significantly fewer cell cycle controls in this microrganism [29,55]. By contrast, the phenotypic groups that have emerged from the collection of conditional growth mutants described in this study support the notion of specific mechanisms and checkpoints. For example, two *ts* mutants were isolated that reversibly arrest in the G1 phase when shifted to 40°C (mutant 63H4 and 31F1). The presence of such a natural G1 checkpoint is further supported by the observation that end-stage differentiated parasite forms (sporozoite and bradyzoite) show a uniform haploid DNA content [28,56], as do parasites that have been treated with the G1 phase inhibitor pyrrolidine dithiocarbamate [57,58]. Tachyzoites released from this drug block, grow synchronously through at least two division cycles, indicating that pyrrolidine dithiocarbamate is likely acting on the same G1 checkpoint affected in our mutants.

Another important checkpoint controls entry into S phase, and in *Saccharomyces*, this checkpoint (called START) also controls the initiation of spindle formation and budding [59]. We have previously argued that the tachyzoite cell cycle likely has a similar checkpoint based on the observation that dNTP depletion arrests tachyzoite growth at the G1/S boundary (1N DNA content, centrosomes largely duplicated but not yet separated [22,28]). We have isolated five *ts* mutants (*e.g.* mutant 150B8) that display a very similar phenotype at the restrictive temperature, suggesting that the G1/S transition is an important restriction point in the tachyzoite cell cycle as it is in yeast.

Mitosis in Apicomplexa has several unique features: the nucleus remains intact, the intranuclear spindle(s) reside in a peculiar elaboration of the nuclear envelope the so called centrocone, and daughter cells are scaffolded as internal buds which develop in close proximity and likely under the control of the extranuclear centrosomes [1,60]. There is significant evidence for the tight regulation of mitotic events in tachyzoites from three groups of mutants in our collection. These mutants arrest either in mitosis (11C9), display defects in chromosome segregation (5 mutants), or loose the coordination of karyokinesis with cytokinesis at various stages in the replication timeline (12 mutants).

Mitotic mutant V-A15 becomes both aneuploid and polyploid at the restrictive temperature and fails to initiate internal budding. The gene affected in this mutant is an NIMA-related serine/threonine kinases (Nek). This kinase family was first identified as essential for division in Aspergilus nidulans [61] and its members have since been identified as cell cycle regulators throughout eukaryotes [62,63], including protozoa [35,36,64]. Neks have roles in microtubular dynamics in cilia, mitotic spindles and centrioles [63,65,66]. Consistent with the known roles for NIMA-related kinases, the mutation in V-A15 leads to defects in the spindle apparatus (reflected in the loss of MORN1 organization) and this causes chromosome mis-segregation. Apicomplexa encode a family of related NEK proteins, and in P. falciparum these genes have been shown to be expressed in a developmentally regulated fashion, making it likely that NEKs are critical to fine-tuning the cell cycle to different life-cycle stages and host cells.

At the non-permissive temperature mutants 42D6 and PO-B3 are promiscuous for nuclear reduplication leading to the formation of syncytial cells with multiple nuclei. Daughter budding is also abnormal in these mutants and uncoupled from the controls that ensure proper nuclear sorting into each daughter. Two distinct RCC1 domain proteins were found to rescue these mutants (25.m01896 and 72.m00409). In other eukaryotes, RCC1 domain proteins interact with Ran-GTPases to regulate spindle assembly as well as other mitotic progression controls through modulation of nuclear trafficking [67]. Like mutant V-A15, mutant PO-B3 (and also uncoupling mutant 42D6) looses MORN1 organization at the restrictive temperature pointing to a potential spindle defect (data not shown). Overall the phenotype of this mutant, as well as several other members of the uncoupling class produced here (*e.g.* 42D6, 20C2, and 7A11), are similar to the abnormal daughter budding and the induction of unregulated nuclear replication associated with the disruption of the parasite spindle by pharmacological microtubule ablation [29]. Collectively, these observations indicate that proper control over chromosome copy number and budding in Apicomplexa might critically rely on an intact intranuclear spindle or associated structures. In this context it is important to note that the unique centrocone structure that conducts the apicomplexan spindle into the nucleus appears to persist throughout the cell cycle at least in some Apicomplexa [20,68]. This model is further supported by preliminary electron microscopy studies of mitotic mutant 11C9, which upon temperature arrest retains an intact spindle and early daughter scaffolds ([32] and S. Halonen and M. White, unpublished data) and does not undergo nuclear reduplication as is seen in mutants 42D6 and PO-B3. Thus, these models predict that rather than the absence of cell cycle controls in the Apicomplexa, mitotic control mechanisms might require a strict physical context associated with the centrosome and/or the centrocone of the parasite nucleus. Breaking this physical context by drug treatment [29], overexpression of a centrocone structural componenent [20], or mutation (as in the uncoupling group of *ts* mutants) results in catastrophic loss of regulation. There is considerable precedence for spatial control of cell cycle checkpoint proteins through compartmental exclusion as well as physical tethering of factors to the centrosomes and spindle structure [69]. Strict compartmentalization of the cell cycle machinery could be a key to the cell cycle flexibility observed in these parasites. Further work is needed to validate this hypothesis. However, the large collection of mutants and genes identified in this screen provides an important pool of validated candidates for mechanistic dissection. Studies that link parasite cell cycle control to the adaptation to specific host cell niches and pathogenesis will be of particular interest.

## Materials and Methods

### High-throughput isolation of temperature-sensitive mutants.

Parasites were grown in human foreskin fibroblasts (HFF) as described [70]. All transgenic and mutant parasite lines are derivatives of the RH parasite line.

The non-fluorescent, visually screened mutants were generated from a RH-*hxgprt-*parasites parent line. Parasites grown overnight at 37°C in 150 mm plates are mutagenized with 400 μg/ml ethyl-nitroso-urea (ENU) for 4 h at 37°C (∼70% killing) [9]. The plates were washed to remove the mutagen and the parasites harvested by needle-passage and purified by filtration. Purified parasites are diluted to 40 parasites/ml and 0.15 ml/well plated directly into 96-well plates, which yields ∼33 single plaques per plate line (Poisson distribution predicts 37.5% maximum). Following 10 days of growth, the master plates are scored for single colonies and then replica plated into two test plates (34°C and 40°C) using a 96-pin array that transfers 20 μl per pin (VP 403E Grooved Pin replicator, V&P Scientific) [13]. Growth/no-growth at 34°C and 40°C is scored 7 days later and putative *ts* mutants are passed into duplicate 24-well plates to reconfirm temperature-sensitive phenotype (secondary test). Each screen of 200–96-well plates yielded ∼6,000 single ENU clones (see Table S1).

Cytoplasmic YFP expressing parasite mutants are based on the 2F-1 YFP2 line parent line [13]. In addition, these parasites stably express β-galactosidase. Chemical mutagenesis was performed as described above. Following mutagenesis and release from the host cells, parasites were resuspended in medium without phenol red at a concentration of 250 parasites/ml and cloned directly in 384-well plates (transparent TC-coated plates; NUNC, Roskilde, Denmark) confluent with HFF cells using a plate filler at 40 μl/well (Q-fill: Genetics, New Milton, UK). Parasites were expanded for 7 days at 35°C. To suspend parasites, the plates were shaken 1 min at maximum speed on a Bellco orbital shaker (Vineland, NJ) placed inside a laminar flow hood. Parasites were grown for six more days until approximately 80% of the monolayer was lysed. Plates were shaken again and duplicated into two identical copies in black-with-optical-bottom TC treated 384-well plates (Falcon/BD, San Jose, CA) using a custom-made 384-pin tool with 5 μl slots (VP Scientific, San Diego, CA). Plate copies were preseeded with HFF cells and 40 μl of medium without phenol red. One plate was kept at 35°C and the second copy was incubated at 40°C. After 4 days YFP fluorescence was measured in each well using a FluoStar plate reader (BMG, Offenburg, Germany) as described [13]. Differential relative fluorescence units of wells in the 35°C versus the 40°C plate were calculated using a script in Excel (Microsoft, Redmond, WA). All identified *ts* clones were expanded into T25 flasks at 35°C and their growth arrest at 40°C was confirmed by microscopy in a 24-well plate format.

### Phenotypic analysis of *ts* mutants.

Parasite reversion frequencies were measured by plaque assay at 34°C versus 40°C using several parasite dilutions (10^7^-10^4^ parasites/T175cm^2^ flask).

DNA content and cellular and nuclear morphologies at the permissive and restrictive temperatures were used to characterize the phenotypes of individual *ts*-clones. A culture of parasites in log phase (8–16 parasites per vacuoles) was used to seed duplicate T75cm^2^ flasks (5x10^6^ parasites/flask) for Sytox-Green flow cytometry and to inoculate 6 well plates with coverslips (1x10^6^ parasites/well) for immunofluorescence assays. Following a 2 h incubation at 34°C, the cultures were washed 3x, allowed to grow at 34°C for 5 h and then one set of cultures was shifted to 40°C.

Co-staining of infected cultures with a monoclonal mouse antibody 45.15 for IMC1 (1:2000, a gift from Dr. Ward, University of Vermont) and a polyclonal rabbit antiserum for TgPCNA1 (1:5000) [71] was used to examine changes in cell morphologies and to determine the presence of internal daughter buds and to establish whether replication foci indicative of active DNA replication were present. Where indicated, further antibodies used are: monoclonal 20H5 specific for centrin1 (1:2000; a generous gift from Dr. Salisbury (Mayo Clinic, Rochester, MN) [72]; rabbit anti-TgMORN1 (1:200) [20]; rabbit anti-TgIMC3 (1:1000) [25]; rabbit anti-GFP (1:5000; Torrey Pines Biolabs, CA); monoclonal antibody 12G10 anti-α-tubulin (1:10; a kind gift from Jacek Gaertig Univ. of Georgia, [73]). 4',6-diamidino-2-phenylindole (DAPI) staining was also performed to judge the qualitative changes in DNA content in parasite clones growth at 34°C versus 40°C. Briefly, parasite cultures grown on coverslips in 6-well plates were fixed with 3.7% paraformaldehyde (pH7.4) and then permeabilized with 0.25% Triton X-100. Coverslips were washed and incubated in 1X PBS pH 7.4 containing 5% FBS and 3% BSA (blocking solution) for at least 30 min. Primary antibodies diluted in blocking solution were incubated for 30–60 min, the coverslips washed 3X with blocking buffer and then incubated for 30–60 min with secondary antibodies: Alexa Fluor 594 for mouse and Alexa Fluor 488 for rabbit diluted 1:2000 in blocking buffer. The secondary antibodies were removed and a DAPI solution (1 mg/ml stock) diluted 1:100 in blocking buffer was added for 5–10 min prior to washing and mounting the coverslips in gel mount. Parasites are evaluated with an epifluorescence microscope (Eclipse TE300, Nikon Inc., Melville NY) and images collected with a digital camera (SPOT^TM^, Dynamic Instruments Inc). In addition, pictures were acquired on a DM IRB inverted microscope (Leica) equipped with a PLAPO 100x/1.4 lens and as well as an Axiovert 200M inverted microscope (Zeiss) equipped with a PRIOR stage and a NEOFLUAR 100x/1.3 lens. The latter two microscopes are equipped with a Hamamatsu C4742–95 CCD camera, and both are controlled by Improvision software (Lexington, MA).

### Flow cytometric determination of nuclear DNA content.

Parasite nuclear DNA content was evaluated by flow cytometry using SYTOX Green (Invitrogen) staining of tachyzoites grown at the permissive and restrictive temperatures [2]. Briefly, parasites were harvested from the T75cm^2^ flasks by needle passage and filtration through 3 μm filters. Parasites collected by centrifugation were resuspended in 300 μl cold PBS and 700 μl of cold 100% ethanol added drop-wise. The fixed samples were stored at −20°C for at least 24 h prior to staining for flow cytometry. Fixed parasites are pelleted at 3000 x *g*, resuspended in 50 μM Tris pH 7.5 at a final concentration of 6 × 10^6^ parasites/ml and stained with SYTOX Green (1 μM). RNase cocktail (250 U; RNase A, RNase T1) was added and the parasites incubated in the dark at room temperature for 30 min. Nuclear DNA content was measured based on fluorescence (FL-1) using a 488 nm argon laser flow cytometer. Fluorescence was collected in linear mode (10,000 events) and the results were quantified using CELLQuest^TM^ v3.0 (Becton-Dickinson Inc.). The percentages of each cell cycle phase were calculated based on defined gates for each population.

### Establishment of a genomic cosmid library.

A cosmid library was constructed in ToxoSuperCos. This new cosmid backbone plasmid is composed of the double cos-sites from plasmid SuperCos (Stratagene, La Jolla, CA) combined with the kanamycin resistance gene and the origin of replication from pDONR201 (Invitrogen, Carlsbad, CA), the drug pyrimethamine resistant allele of T. gondii dihydrofolate reductates-thymidylate synthase (DHFR-TSm2m3) from pDHFR-TSc3ABP [31] and a synthetic multiple cloning site containing restriction sites for *Kpn*I, *Hin*dII, *Bgl*II and *Xho*I. Briefly, pDONR201 was digested with *Pst*I, ends were blunted with T4 DNA Polymerase and digested with *Apa*I. The resulting 2041 bp fragment was ligated with the DHFR-TSm2m3 expression cassette obtained from pDHFR-TSc3ABP by *Not*I digestion, T4 DNA polymerase treatment and *Apa*I digestion as described above. Subsequently, the double cos-sites from SuperCos were introduced in the pDONR-DHFR-TS hybrid by digesting this plasmid with *Pac*I and *Apa*I and ligation with a PCR product containg the cos-sites (F-primer(*Apa* I): ACTGGGGCCCCGTCTTCAAGAATTCGC; R-primer(*Pac*I): ACTGTTAATTAAAGGCTCTCAAGGGCATCGG) using SuperCos as a template digested with *Pac*I and *Apa*I. Lastly, a polylinker was introduced into the *Spe*I site by hybridizing two synthetic oligonucleotides: F-hyb: CTAGTGGTACCAAGCTTAGATCTCTCGAGA and R-hyb: CTAGTCTCGAGTGATCTAAGCTTGGTACCA followed by ligation into the plasmid opened by *Spe*I restriction. The resulting ToxoSuperCos plasmid is shown in Figure 5.

To construct a cosmid library, genomic DNA was extracted from wild type RH strain. Parasites were lysed in 0.2% SDS in 10 mM Tris/HCl pH 8.0 followed by digestion with 0.8 μg/ml proteinase K and 0.04 μg/ml RNaseA for 4 hrs at 37°C. DNA was extracted once with phenol-chloroform-isoamylalchohol (25:24:1) followed by chloroform extraction and precipitation using 0.2 volumes of 10 M NH_4_OAc and 2 volumes of 100% ethanol. DNA was spooled out using a glass rod and allowed to resuspend in two volumes of TE overnight at 4°C. 5–10 μl of this DNA preparation was digested with a serial dilution of *Sau*3AI. Reactions were analyzed on 1% agarose gels (0.5xTBE) by pulsed field gel electrophoreses on a BioRad CHEF system (15 hrs at 4°C using conditions optimized for separation from 5–200 kb at a gradient of 6.0 V/cm and an angle of 120°) using the New England Biolabs (Beverly, MA) Low Range PFGE marker as size standard. Conditions generating the highest amount of fragments between 40–55 kb (as well as one dilution step above and below) were chosen for preparative *Sau*3AI digestion. After verification by CHEF, the three digests were pooled and treated as described in the SuperCos manual (Stratagene). Briefly, digested genomic DNA was phenol extracted, treated with calf intestinal phosphatase (CIP) and phenol extracted again. The ToxoSuperCos plasmid was prepared by digestion with *Xba*I, CIP treatment and *Bam*HI digestion. Genomic DNA (2.5 μg) and plasmid (1 μg) were ligated overnight and packaged with the Gigapack III XL packaging extract (Stratagene) selecting for inserts between 47 and 51 kb. Cosmid-Phages were infected into XL-blue MRF' E. coli and plated on 10 μg/ml LB-kanamycin plates. The resulting library had a complexity of 1.25 x10^6^ independent colonies and was frozen as a bacterial stabilate.

### Complementation of T. gondii mutants by cosmid transfection.

To produce cosmid library DNA for parasite complementation transfections the library was amplified by plating the stabilate at a density of 10^4^ colonies per 150 mm diameter Petri dishes (10 μg/ml LB-Kan; 50 dishes per batch), colonies were scraped and cosmid DNA was isolated using the Qiagen (Hilden, Germany) Large-Construct Kit according to the manufacturer's instructions. 25 μg cosmid DNA was used for electroporation of 8 x10^7^ parasites prior to inoculation into T175cm^2^ flasks. Parasites were allowed to recover for 24 hrs at 35°C prior to shifting the to 40°C and addition of 1 μM pyrimethamine to select for phenotypic complementation and cosmid backbone genomic integration, respectively. Individual cosmids from the tiled and sequenced plates were isolated from 5 ml bacterial cultures. Cells were lysed using 250 μl P1 buffer (Qiagen) supplemented with 70 μl 10 mg/ml lysozyme solution, followed by addition of Qiagen bufferes P1 and P3 according to the manufacturer's instructions. Following centrifugation, cosmid DNA was precipitated from the supernatant by addition of 0.7 volumes of isopropanol.

### Identification of complementing cosmids by plasmid rescue.

Genomic DNA was extracted from complemented parasite lines (polyclonal or clonal lines) and 2 μg of DNA was digested with one of the restriction enzymes in the linker region (20 U *Xho*I, *Hin*dIII, *Bgl*II or *Kpn*I). Digested DNA was purified using the Quiaquick PCR purification kit (Qiagen) and an equivalent of 0.3 μg digested DNA was self-ligated (overnight) in a volume of 20 μl at 16°C. After phenol extraction and ethanol precipitation, 15% of the total reaction (1.5 μl of 10 μl) was electroporated into 20 μl DH12S electromax E. coli (Invitrogen) and plated on LB-KAN (50 μg/ml, DNA can also be purified using a Qiagen spin column instead of phenol extraction). DNA minipreparations were isolated for multiple colonies and digested with *Bgl*II/*Not*I. Inserts were sequenced using the T3 promoter primer.

## Supporting Information

Figure S1 Transfection of HXGPRT KO Mutant with the ToxoSuperCos Library Results in Complementation of the HXGPRT Locus The RH HXGPRT knock out line was transfected with the ToxoSuperCos library and selected with mycophenolic acid for restoration of HXGPRT activity. Five out of five transfections resulted in stable drug resistant parasites. Clonal lines were established from three transfections and parasites were tested for the presence of diagnostic PCR products for the intact wild type gene ([B], 0.48 kb, minor bands were sequenced and proved to be unrelated spurious amplification products, asterisks) and the KO locus ([C], 0.7 kb). Note that restoration was confirmed in 10 out of 15 clones. Persistence of the KO locus suggests that this did not occur through homologous recombination. (A) Shows a simplified partial map of the HXGPRT locus indicating the positions of primers used.
(2.5 MB TIF)

Figure S2 Expression Level and Size of Complemented Genes (A) Most of the genes identified as cell cycle regulators by complementation show modest expression judged by replicate (average of three independent samples) microarray analysis of Type I strain tachyzoites using the Affymetrix *Toxoplasma* GeneChip (see ToxoDB) (RMA normalized flourescent intensities are shown in red and ranked by expression level: 50.m0307, 52.m01560, 41.m01273, 44.m02615, 583.m0547, 25.m01896, 27.m00873, 35.m00891, 44.m0278, 80.m02355, 20.m03745, 72.m00409, 20.m03766, 49.m00063; see Table 1 for complementation details). The mRNA level for well-characterized genes in the high (GRA1 and TUB) and moderate (PCNA) abundance classes are shown for comparison in blue.
(B) Complemented genes are of varying size (the size of the predicted protein product in number of amino acids is shown here) including numerous large genes. Note that these numbers are based on the current draft three annotation of the T. gondii genome (http://www.toxodb.org/) and that the gene models have not been experimentally validated. The order of genes is the same as in (A).
(193 KB TIF)

Table S1 Summary of ENU Screens (Note That *ts* Mutants Obtained from the YFP Fluorescent Screen Are Indicated by –Y) (31 KB DOC)

Table S2 Summary of the Cell Cycle Analysis of Selected *ts* Mutants (147 KB DOC)

## Abbreviations

ENU: *N*-nitroso-*N*-ethylurea
IFA: immunofluorescence assay
IMC: inner membrane complex
MORN: membrane occupation recognition nexus
PCNA: proliferating cell nuclear antigen
*ts*: temperature sensitive
YFP: yellow fluorescent protein

## References

[ppat-0040036-b001] Striepen B, Jordan CN, Reiff S, van Dooren GG (2007). Building the perfect parasite: cell division in apicomplexa. PLoS Pathog.

[ppat-0040036-b002] White MW, Radke JA, Conde de Felipe M, Lehmann M, Ajioka JW, Soldati D (2007). Cell cycle control/parasite division. The biology of Toxoplasma gondii.

[ppat-0040036-b003] Chen Y, Jirage D, Caridha D, Kathcart AK, Cortes EA (2006). Identification of an effector protein and gain-of-function mutants that activate Pfmrk, a malarial cyclin-dependent protein kinase. Mol Biochem Parasitol.

[ppat-0040036-b004] Khan F, Tang J, Qin CL, Kim K (2002). Cyclin-dependent kinase TPK2 is a critical cell cycle regulator in Toxoplasma gondii. Mol Microbiol.

[ppat-0040036-b005] Kvaal CA, Radke JR, Guerini MN, White MW (2002). Isolation of a Toxoplasma gondii cyclin by yeast two-hybrid interactive screen. Mol Biochem Parasitol.

[ppat-0040036-b006] Ward P, Equinet L, Packer J, Doerig C (2004). Protein kinases of the human malaria parasite Plasmodium falciparum: the kinome of a divergent eukaryote. BMC Genomics.

[ppat-0040036-b007] Black MW, Arrizabalaga G, Boothroyd JC (2000). Ionophore-resistant mutants of Toxoplasma gondii reveal host cell permeabilization as an early event in egress. Mol Cell Biol.

[ppat-0040036-b008] Pfefferkorn ER, Pfefferkorn LC (1976). Toxoplasma gondii: isolation and preliminary characterization of temperature-sensitive mutants. Exp Parasitol.

[ppat-0040036-b009] Radke JR, Guerini MN, White MW (2000). Toxoplasma gondii: characterization of temperature-sensitive tachyzoite cell cycle mutants. Exp Parasitol.

[ppat-0040036-b010] Uyetake L, Ortega-Barria E, Boothroyd JC (2001). Isolation and characterization of a cold-sensitive attachment/invasion mutant of Toxoplasma gondii. Exp Parasitol.

[ppat-0040036-b011] Donald RGK, Carter D, Ullman B, Roos DS (1996). Insertional tagging, cloning, and expression of the Toxoplasma gondii hypoxanthine-xanthine-guanine phosphoribosyltransferase gene. Use as a selectable marker for stable transformation. J Biol Chem.

[ppat-0040036-b012] Pfefferkorn ER, Borotz SE (1994). Toxoplasma gondii: characterization of a mutant resistant to 6- thioxanthine. Exp Parasitol.

[ppat-0040036-b013] Gubbels MJ, Li C, Striepen B (2003). High-throughput growth assay for Toxoplasma gondii using yellow fluorescent protein. Antimicrob Agents Chemother.

[ppat-0040036-b014] Radke JR, Guerini MN, White MW (2000). Toxoplasma gondii: Characterization of Temperature-Sensitive Tachyzoite Cell Cycle Mutants. Exp Parasitol.

[ppat-0040036-b015] Simchen G (1978). Cell cycle mutants. Annu Rev Genet.

[ppat-0040036-b016] Hirschberg J, Marcus M (1982). Isolation by a replica-plating technique of Chinese hamster temperature-sensitive cell cycle mutants. J Cell Physiol.

[ppat-0040036-b017] Salisbury JL (1995). Centrin, centrosomes, and mitotic spindle poles. Curr Opin Cell Biol.

[ppat-0040036-b018] Striepen B, Crawford MJ, Shaw MK, Tilney LG, Seeber F (2000). The plastid of Toxoplasma gondii is divided by association with the centrosomes. J Cell Biol.

[ppat-0040036-b019] Hu K, Johnson J, Florens L, Fraunholz M, Suravajjala S (2006). Cytoskeletal components of an invasion machine–the apical complex of Toxoplasma gondii. PLoS Pathog.

[ppat-0040036-b020] Gubbels MJ, Vaishnava S, Boot N, Dubremetz JF, Striepen B (2006). A MORN-repeat protein is a dynamic component of the Toxoplasma gondii cell division apparatus. J Cell Sci.

[ppat-0040036-b021] Guerini M, Que X, Reed SL, White MW (2000). Two genes encoding unique proliferating-cell-nuclear antigens are expressed in Toxoplasma gondii. Mol Biochem Parasitol.

[ppat-0040036-b022] Radke JR, Striepen B, Guerini MN, Jerome ME, Roos DS (2001). Defining the cell cycle for the tachyzoite stage of Toxoplasma gondii. Mol Biochem Parasitol.

[ppat-0040036-b023] Hu K, Mann T, Striepen B, Beckers CJ, Roos DS (2002). Daughter cell assembly in the protozoan parasite Toxoplasma gondii. Mol Biol Cell.

[ppat-0040036-b024] Mann T, Beckers C (2001). Characterization of the subpellicular network, a filamentous membrane skeletal component in the parasite Toxoplasma gondii. Mol Biochem Parasitol.

[ppat-0040036-b025] Gubbels MJ, Wieffer M, Striepen B (2004). Fluorescent protein tagging in Toxoplasma gondii: identification of a novel inner membrane complex component conserved among Apicomplexa. Mol Biochem Parasitol.

[ppat-0040036-b026] Hartwell LH, Mortimer RK, Culotti J, Culotti M (1973). Genetic control of the cell division cycle in yeast: V. genetic analysis of cdc mutants. Genetics.

[ppat-0040036-b027] Hartwell LH (1991). Twenty-five years of cell cycle genetics. Genetics.

[ppat-0040036-b028] Radke JR, White MW (1998). A cell cycle model for the tachyzoite of Toxoplasma gondii using the Herpes simplex virus thymidine kinase. Mol Biochem Parasitol.

[ppat-0040036-b029] Morrissette NS, Sibley LD (2002). Disruption of microtubules uncouples budding and nuclear division in Toxoplasma gondii. J Cell Sci.

[ppat-0040036-b030] Radke JR, Gubbels MJ, Jerome ME, Radke JB, Striepen B (2004). Identification of a sporozoite-specific member of the Toxoplasma SAG superfamily via genetic complementation. Mol Microbiol.

[ppat-0040036-b031] Striepen B, White MW, Li C, Guerini MN, Malik SB (2002). Genetic complementation in apicomplexan parasites. Proc Natl Acad Sci U S A.

[ppat-0040036-b032] White MW, Jerome ME, Vaishnava S, Guerini M, Behnke M (2005). Genetic rescue of a Toxoplasma gondii conditional cell cycle mutant. Mol Microbiol.

[ppat-0040036-b033] Donald RG, Roos DS (1993). Stable molecular transformation of Toxoplasma gondii: a selectable dihydrofolate reductase-thymidylate synthase marker based on drug- resistance mutations in malaria. Proc Natl Acad Sci U S A.

[ppat-0040036-b034] Roos DS, Sullivan WJ, Striepen B, Bohne W, Donald RG (1997). Tagging genes and trapping promoters in Toxoplasma gondii by insertional mutagenesis. Methods.

[ppat-0040036-b035] Dorin D, Le Roch K, Sallicandro P, Alano P, Parzy D (2001). Pfnek-1, a NIMA-related kinase from the human malaria parasite Plasmodium falciparum Biochemical properties and possible involvement in MAPK regulation. Eur J Biochem.

[ppat-0040036-b036] Reininger L, Billker O, Tewari R, Mukhopadhyay A, Fennell C (2005). A NIMA-related protein kinase is essential for completion of the sexual cycle of malaria parasites. J Biol Chem.

[ppat-0040036-b037] Oakley BR, Morris NR (1983). A mutation in Aspergillus nidulans that blocks the transition from interphase to prophase. J Cell Biol.

[ppat-0040036-b038] O'Regan L, Blot J, Fry AM (2007). Mitotic regulation by NIMA-related kinases. Cell Div.

[ppat-0040036-b039] Bauer A, Kolling R (1996). The SAC3 gene encodes a nuclear protein required for normal progression of mitosis. J Cell Sci.

[ppat-0040036-b040] Halawani D, Latterich M (2006). p97: The cell's molecular purgatory. Mol Cell.

[ppat-0040036-b041] Cheeseman IM, Desai A (2004). Cell division: AAAtacking the mitotic spindle. Curr Biol.

[ppat-0040036-b042] Minoda A, Saitoh S, Takahashi K, Toda T (2005). BAF53/Arp4 homolog Alp5 in fission yeast is required for histone H4 acetylation, kinetochore-spindle attachment, and gene silencing at centromere. Mol Biol Cell.

[ppat-0040036-b043] Itoh T, Satoh M, Kanno E, Fukuda M (2006). Screening for target Rabs of TBC (Tre-2/Bub2/Cdc16) domain-containing proteins based on their Rab-binding activity. Genes Cells.

[ppat-0040036-b044] Frankel MB, Mordue DG, Knoll LJ (2007). Discovery of parasite virulence genes reveals a unique regulator of chromosome condensation 1 ortholog critical for efficient nuclear trafficking. Proc Natl Acad Sci U S A.

[ppat-0040036-b045] Kaplan Y, Kupiec M (2007). A role for the yeast cell cycle/splicing factor Cdc40 in the G1/S transition. Curr Genet.

[ppat-0040036-b046] Hartwell LH, Culotti J, Reid B (1970). Genetic control of the cell-division cycle in yeast. I. Detection of mutants. Proc Natl Acad Sci U S A.

[ppat-0040036-b047] Talavera A, Basilico C (1977). Temperature sensitive mutants of BHK cells affected in cell cycle progression. J Cell Physiol.

[ppat-0040036-b048] Eki T, Enomoto T, Miyajima A, Miyazawa H, Murakami Y (1990). Isolation of temperature-sensitive cell cycle mutants from mouse FM3A cells. Characterization of mutants with special reference to DNA replication. J Biol Chem.

[ppat-0040036-b049] Black MW, Boothroyd JC (1998). Development of a stable episomal shuttle vector for Toxoplasma gondii. J Biol Chem.

[ppat-0040036-b050] Hartley JL, Temple GF, Brasch MA (2000). DNA cloning using in vitro site-specific recombination. Genome Res.

[ppat-0040036-b051] Taylor S, Barragan A, Su C, Fux B, Fentress SJ (2006). A secreted serine-threonine kinase determines virulence in the eukaryotic pathogen Toxoplasma gondii. Science.

[ppat-0040036-b052] Clarke DJ, Gimenez-Abian JF (2000). Checkpoints controlling mitosis. Bioessays.

[ppat-0040036-b053] Huberman JA (1996). Cell cycle control of S phase: a comparison of two yeasts. Chromosoma.

[ppat-0040036-b054] Murray JM, Hunter CA (1993). The cell cycle: an introduction.

[ppat-0040036-b055] Shaw MK, Roos DS, Tilney LG (2001). DNA replication and daughter cell budding are not tightly linked in the protozoan parasite Toxoplasma gondii. Microbes Infect.

[ppat-0040036-b056] Radke JR, Guerini MN, Jerome M, White MW (2003). A change in the premitotic period of the cell cycle is associated with bradyzoite differentiation in Toxoplasma gondii. Mol Biochem Parasitol.

[ppat-0040036-b057] Moon SK, Jung SY, Choi YH, Lee YC, Patterson C (2004). PDTC, metal chelating compound, induces G1 phase cell cycle arrest in vascular smooth muscle cells through inducing p21Cip1 expression: involvement of p38 mitogen activated protein kinase. J Cell Physiol.

[ppat-0040036-b058] Conde de Felipe MM, Lehmann MM, Jerome ME, White MW (2008). Inhibition of Toxoplasma gondii growth by pyrrolidine dithiocarbamate is cell cycle specific and leads to population synchronization. Mol Biochem Parasitol.

[ppat-0040036-b059] Hartwell LH, Culotti J, Pringle JR, Reid BJ (1974). Genetic control of the cell division cycle in yeast. Science.

[ppat-0040036-b060] Ferguson DJP, Dubremetz JF, Weiss LM, Kim K (2007). The ultrastructure of *Toxoplasma gondii*.

[ppat-0040036-b061] Osmani SA, May GS, Morris NR (1987). Regulation of the mRNA levels of nimA, a gene required for the G2-M transition in Aspergillus nidulans. J Cell Biol.

[ppat-0040036-b062] Letwin K, Mizzen L, Motro B, Ben-David Y, Bernstein A (1992). A mammalian dual specificity protein kinase, Nek1, is related to the NIMA cell cycle regulator and highly expressed in meiotic germ cells. Embo J.

[ppat-0040036-b063] O'Connell MJ, Krien MJ, Hunter T (2003). Never say never. The NIMA-related protein kinases in mitotic control. Trends Cell Biol.

[ppat-0040036-b064] Pradel LC, Bonhivers M, Landrein N, Robinson DR (2006). NIMA-related kinase TbNRKC is involved in basal body separation in Trypanosoma brucei. J Cell Sci.

[ppat-0040036-b065] Quarmby LM, Mahjoub MR (2005). Caught Nek-ing: cilia and centrioles. J Cell Sci.

[ppat-0040036-b066] Wloga D, Camba A, Rogowski K, Manning G, Jerka-Dziadosz M (2006). Members of the NIMA-related kinase family promote disassembly of cilia by multiple mechanisms. Mol Biol Cell.

[ppat-0040036-b067] Kalab P, Pu RT, Dasso M (1999). The ran GTPase regulates mitotic spindle assembly. Curr Biol.

[ppat-0040036-b068] Vaishnava S, Morrison DP, Gaji RY, Murray JM, Entzeroth R (2005). Plastid segregation and cell division in the apicomplexan parasite Sarcocystis neurona. J Cell Sci.

[ppat-0040036-b069] Pines J (1999). Four-dimensional control of the cell cycle. Nat Cell Biol.

[ppat-0040036-b070] Roos DS, Donald RG, Morrissette NS, Moulton AL (1994). Molecular tools for genetic dissection of the protozoan parasite Toxoplasma gondii. Methods Cell Biol.

[ppat-0040036-b071] Guerini MN, Behnke MS, White MW (2005). Biochemical and genetic analysis of the distinct proliferating cell nuclear antigens of Toxoplasma gondii. Mol Biochem Parasitol.

[ppat-0040036-b072] Sanders MA, Salisbury JL (1994). Centrin plays an essential role in microtubule severing during flagellar excision in Chlamydomonas reinhardtii. J Cell Biol.

[ppat-0040036-b073] Jerka-Dziadosz M, Frankel J (1995). The effects of lithium chloride on pattern formation in Tetrahymena thermophila. Dev Biol.

