# Supplementary material for: Forward Genetic Analysis of the Apicomplexan Cell Division Cycle in Toxoplasma gondii
Source: PLoS Pathog. 2008 Feb 15;4(2):e36. doi: 10.1371/journal.ppat.0040036 (PMC2242837; doi:10.1371/journal.ppat.0040036)
Supplement: Supplementary file 1 [file ppat.0040036.st001.doc]

**Table S1: Summary of ENU screens (note that *ts*-mutants obtained from the**

YFP fluorescent screen are indicated by –Y)

| **Screen** | **Total ENU**  **single colonies** | **Preliminary**  ***ts* calls** | ***ts* phenotype**  **confirmed** |
| --- | --- | --- | --- |
| **Visual screen**  **13 exps** | **32,636** | **2,020** | **113**  **(0.35%)** |
| **YFP screen**  **22 exps** | **~30,000** | **940** | **52**  **(0.17%)** |
| **Total** | **62,636** | **2,960** | **165**  **(0.26%)** |
