# Supplementary material for: Forward Genetic Analysis of the Apicomplexan Cell Division Cycle in Toxoplasma gondii
Source: PLoS Pathog. 2008 Feb 15;4(2):e36. doi: 10.1371/journal.ppat.0040036 (PMC2242837; doi:10.1371/journal.ppat.0040036)
Supplement: Supplementary file 2 [file ppat.0040036.st002.doc]

**Table S2. Summary of the cell cycle analysis of selected *ts*** mutants.

| **Ph**  **ase arrest** | **Phenotypic criteria for cell cycle classifications.** | | | | |
| --- | --- | --- | --- | --- | --- |
| G1 phase arrest | (1) Predominant 1N DNA content (>75% of the population) by FACS analysis.  (2) Single nucleus per parasite and G1 phase TgPCNA1 staining pattern in >75% of the population. PCNA1 staining patterns show a small nucleus with dull, uniform fluorescence intensity.  (3) No internal IMC1 daughter forms in >75 % of the population. | | | | |
| S Phase arrest | (1) Genomic DNA distributions by FACS analysis show major subpopulations with >1N, but <2N DNA contents.  (2) Large and centrally located single nucleus with bright , punctateTgPCNA1 staining pattern.  (3) Internal daughters are usually absent, or if present, they are arrested at an early stage of formation (late S phase pattern). | | | | |
| Mitotic arrest | (1) Genomic DNA distributions by FACS analysis show major subpopulations with ≥2N DNA contents.  (2) Daughter to nuclei ratios is in the normal range (1:1, 2:1, 2:2).  (3) TgPCNA1 staining patterns are more homogenous and dull. | | | | |
| Chromsome mis-segregation | (1) Genomic DNA distributions by FACS show major subpopulations with <1N DNA content.  (2) Evidence of nuclear fragmentation in dapi and TgPCNA1 staining patterns.  (3) Evidence of daughter parasites containing no or little dapi material (zoid formation). | | | | |
| Budding mutants (early or late) | (1) Evidence of growth arrest with discernable IMC1 staining structures. Early mutant may have small intense IMC1 body, while late mutant would have more extensive development of the daughter bud.  (2) Nucleus daughter ratios fall within the normal range indicating nuclear segregation is occurring.  (3) No evidence of formation of multinucleate syncytial cells. | | | | |
| Uncoupling mutants | (1) Abnormal daughter to nuclear ratios. Formation of syncytial cells with multiple nuclei  (2) Multiple IMC1 structures.  (3) Genomic DNA distributions are heterogeneous, with evidence of polyploidy. | | | | |
| **Summary of phenotypic features for each *ts* mutant analyzed in the clone collection.** | | | | | |
|  | | | | **Key arrest phenotypes** | |
| **Mutant Class** | **Clone label** | **Number of 40^o^C divisions & vacuole size at arrest.** | **Revertant frequency** | **Cell biology of 40^o^C growth arrest.** | **FACS DNA profiles at 40^o^C growth arrest.** |
| **G1/G0 mutants Subclass 1:** Loss of nuclear TgPCNA1 staining. | 124H2 | 2-3 divisions/ 4 & 8 parasites per vacuole | <10-7 | 1) normal size and shape. 2) nucleus:parasite ratio=1:1.  3) no internal daughters,  4) unusual loss of nuclear PCNA1 staining--low expression and no nuclear concentration. Note that this pattern is seen in dormant sporozoites & mature bradyzoites (not shown) | 1) predominant 1N subpopulation.  2) small 2N subfraction (<10%), which is due in part to parasites that contain double nuclei but failed to bud. |
| Loss of TgPCNA1 nuclear staining | 133D12 | 0-2 divisions/ 1, 2, & 4 parasites per vacuole | <10-7 | 1) normal shape and size; small parasites consistent with G1 phase.  2) nucleus:parasite ratios 1:1.  3) very few internal daughters.  4) PCNA1 staining is diminished and no longer concentrated in the nucleus. There is some PCNA1 staining in the vacuole space.  (This phenotype is very similar to mutant #24 12-124H2). | 1) predominant 1N subpopulation.  2) ~20% 2N subfraction due in part to parasites that contain double nuclei but failed to bud. |
|  | | | | | |
| **G1 mutant: Subclass 2:** Uniform G1 phenotype, many arrest quickly with 0-1 divisions | 45G3 | 1-2 divisions/ 2 & 4 parasites per vacuole | <10-7 | 1) normal parasite size and shape.  2) nucleus:parasite ratios=1:1  3) no internal daughters, 4) PCNA1 stain is consistent with G1 nucleus (small nucleus with uniform low intensity fluorescence) | 1) predominant tight 1N population.  2) small amount ~2N (<10%) |
|  | 109C6 | 0-1 divisions/ 1 & 2 parasites per vacuole | <10-7 | 1) normal parasite size and shape.  2) nucleus:parasite ratios=1:1.  3) no internal daughters  4) PCNA1 staining pattern consistent with G1 phase | 1) predominant and uniform 1N DNA content. |
|  | 88A5 | 0-1 divisions/ 1 & 2 parasites per vacuole | <10 -6 | 1) smaller parasite size but normal shape.  2) nucleus:parasite ratio=1:1.  3) no internal budding.  4) PCNA1 staining is a uniform G1 pattern | 1) predominant uniform 1N DNA content. |
|  | 79F1 | 0-2 divisions/ mostly 1 & 2 with a few 4 parasites per vacuole | <10 -6 | 1) small size but normal shape.  2) nucleus:parasite ratio=1:1.  3) few internal daughters.  4) PCNA1 stain is consistent with a G1 nucleus | 1) predominant, broad 1N DNA content. |
|  | 87A10 | 0-2 divisions/ mostly 1 & 2 parasites per vacuole | <10-7 | 1) normal parasite size and shape.  2) nucleus:parasite ratio=1:1.  3) no internal daughters.  4) PCNA1 staining is a uniform G1 pattern. Parasites are very fragile and less stable to harvest after 40^o^C shift | 1) broad, uniform 1N DNA content.  2) considerable debrie in 40^o^C samples indicating parasites die quickly. |
|  | 57H2 | 0-2 divisions/ 1, 2 & 4 parasites per vacuole | <10 -6 | 1) large parasites with near normal shape.  2) nucleus:parasite ratio=1:1.  3) some nuclear division w/o budding, no internal daughters.  4) very prominent plastid by dapi with some plastids deposited in the vacuole.  5) PCNA1 staining pattern is mixed. | 1) broad 1N DNA content. May contain early S phase parasites. |
|  | | | | | |
| **G1 mutant: Subclass 3:** non-lethal, reversible phenotype | 63H4 | 0-1 divisions/ 1 & 2 parasites per vacuole | <10-7 | 1) normal size and shape.  2) nucleus:parasite ratio=1:1.  3) no internal daughters.  4) PCNA1 staining in some parasites is brighter than normal G1 and some nuclei have an elongated shape,  5) Parasites exposed to 24 h at 40^o^C are fully viable. | 1) predominant 1N DNA content, although the 1N peak is broad and may contain a small number of early S phase parasites.  2) small 1.8N subpopulation (<5%). |
|  | 31F1 | 0-1 divisions/ 1 & 2 parasites per vacuole. | <10 -6 | 1) normal size and shape.  2) nucleus:parasite ratio=1:1  3) no internal daughters.  4) PCNA1 staining is largely a normal G1 pattern with ~10% having an S phase pattern. 5) parasites exposed to 24 h at 40^o^C are fully viable. | 1) predominately 1N DNA content.  2) ~10% 1.8N subpopulation. |
|  | | | | | |
| **G1 mutant Subclass 4:** mixed PCNA1 nuclear staining | 37D3 | 1-2 divisions/ 2&4 parasites per vacuole | <10 -6 | 1) large parasite size but normal shape.  2) nucleus:parasite ratios=1:1.  3) no internal daughters. 4) PCNA1 stain is 85% G1 and15% S phase | 1) predominant 1N DNA content.  2) ~15% 1.8N subpopulation. |
|  | 55B1 | 1-2 divisions/ 2&4 parasites per vacuole | <10 -6 | 1) normal parasite size and shape.  2) nucleus:parasite ratio=1:1.  3) no internal daughters. 4) PCNA1 staining is mixed, ~85% G1/~15% S phase. | 1) broad 1 to 1.3N DNA content indicating a small subpopulation of early S phase. |
|  | | | | | |
| **G1 mutant: Subclass 5:** loss of vacuole synchrony | 66B1 | 0-1 divisions/ 1 & 2 parasites per vacuole | <10 -6 | 1) large parasite size and irregular shape.  2) nucleus:parasite ratios=1:1 & 2:1.  3) nuclear division with out budding.  4) unusual loss of vacuole synchrony seen with PCNA1 with G1/S phase patterns in parasites sharing the same vacuole | 1) primary 1N DNA content with some 2N parasites |
|  | | | | | |
| **G1 mutant Subclass 6:** Giant cells, loss of cell volume control | 73C1 | 0-2 divisions/ 1,2,4 parasites per vacuole | <10 -6 | 1) extremely large size and irregular shape in some vacuoles.  2) nucleus:parasite ratio=1:1.  3) no internal daughters.  4) PCNA1 staining is mixed with major G1 and minor S phase patterns. | 1) broad 1N-1.3N DNA content. |
|  | 26C9 | 1-2 divisions/2& 4 parasites per vacuole | <10 -6 | 1) extremely large size and irregular parasite shape in >90% of vacuoles.  2) nucleus:parasite ratio=1:1.  3) no internal daughters.  4) PCNA1 staining is major G1 & minor S phase patterns. | 1) primary 1N DNA content with a ~10% 1.8N subpopulation |
|  |  |  |  |  |  |
| **S phase mutants Subclass 1:** arrest in early to mid S phase | 60G10 | 1-2 divisions/ 2 & 4 parasites per vacuole | <10-7 | 1) distinctive round parasite shape.  2) nucleus:parasite ratio=1:1.  3) no internal daughters.  4) central nucleus with bright, punctate PCNA1 staining pattern. | 1) uniform ~1.3N DNA content. |
|  | 150B10 | 1-2 divisions/ 2 & 4 parasite per vacuole | <10-7 | 1) round parasite shape.  2) nucleus:parasite ratio=1:1.  3) no internal daughters, 4. bright-central PCNA1-stained nucleus consistent with S phase | 1) uniform arrest in early S phase, ~1.3-1.5N DNA content. |
|  | 150B8 | 1-2 divisions/ 2 & 4 parasite per vacuole | <10-7 | 1) uniform large & round parasite shape.  2) nucleus:parasite ratio=1:1.  3) no internal daughters.  4) bright-central PCNA1 staining nucleus consistent with S phase pattern.  5) ~75% of parasites contain duplicated centrosomes. | 1) uniform ~1.3N DNA content. |
|  | 193H1 | 1-2 divisions/ 2 & 4 parasite per vacuole | <10-7 | 1) uniform large & round parasite shape.  2) nucleus:parasite ratio=1:1.  3) no internal daughters.  4) PCNA1 staining is bright and punctate consistent with S phase pattern. | 1) uniform ~1.3N DNA content. |
|  | 60E5 | 0-1 divisions/1 & 2 parasites per vacuole | <10 -6 | 1) extremely large size and irregular shape in >75% of vacuoles.  2) nucleus:parasite ratio is variable with predominant 1:1 ratio.  3) no internal daughters. 4) nuclear morphology by dapi and PCNA1 staining is variable, but consistent with G1 and S phase patterns. | 1) broad 1-1.5N average DNA content with small subpopulation >2N |
|  | P6 | 0-1 divisions/1 & 2 parasites per vacuole | <10 -6 | 1) small parasite size but normal shape.  2) nucleus:parasite ratios=1:1.  3) no internal daughters.  4) PCNA1 stain consistent with minor G1 and major S phase nuclei subpopulations | 1) DNA content distributions span G1 (1N) and early S phase (1.3N) |
|  |  |  |  |  |  |
| **S Phase mutants: Subclass 2:** unusual 1.8N subpopulation | 104A4 | 1-2 divisions/ 2 & 4 parasites per vacuole | <10-7 | 1) unusual S phase mutant, round parasites arrest with 1N and 1.8N content.  2) nucleus:parasite ratio=1:1.  3) few internal daughters,  4) PCNA1 staining consistent with S phase  5) duplicated centrosomes in 50% of parasites | 1) DNA content profile is 50% 1N and 50% 1.8N. |
|  |  |  |  |  |  |
| **Mitotic Mutants Subclass 1:** mis-segregation mutants/ chromosome loss | 51A1 | variable divisions/ odd numbers of parasite per vacuole | <10-7 | 1) irregular parasite shape and size.  2) abnormal daughters.  3) dapi stains show unequal chromosome content with retention of nuclear material in the mother cell.  4) variable nuclear PCNA1 staining. | 1) <1N to 1N DNA content |
|  | 64D5 | 0-2 divisions/ 1, 2, & 4 parasites per vacuole with some odd numbered vacuole | <10 -6 | 1) irregular parasite shape.  2) dapi stain shows nuclear division is unequal leading to chromosome loss. Some nuclei are released free in the vacuole and parasites form without a nucleus (zoids).  3) nuclear PCNA1 staining is highly variable**.** | 1) dominant 1N and sub-1N DNA content. |
|  | 115C5 | variable divisions/ odd numbers of parasite per vacuole | <10 -6 | 1) irregular parasite shapes and variable size.  2) dapi stain shows nuclear division is unequal with formation of zoids.  3) daughter buds fail to resolve mother cell.  4) variable nuclear PCNA1 staining | 1) predominant <1N DNA content indicating chromsome loss |
|  | 83A3 | variable divisions/ odd numbers of parasite per vacuole. | <10-7 | 1) irregular parasite shapes & size.  2) dapi stain shows nuclear division is unequal.  3) daughters form without mother cell resolution. Some daughters are missing a nucleus (zoid), while the mother cell retains a nucleus.  4) Plastids are uncoupled from daughter formation and are lost to the vacuole.  5) nuclear PCNA1 staining is variable. | 1) Predominant <1N DNA content indicating chromosome loss. |
|  | V-A15 | variable divisions/ odd numbers of parasite per vacuole. | <10 -6 | 1) irregular parasite size and shape.  2) dapi stain shows fragmented nuclei.  3) no internal daughters.  4) nuclear PCNA1 staining is heterogeneous. | 1) complex DNA profile by FACS with nearly equal <1N, 1.8N and >3N subpopulations. |
|  |  |  |  |  |  |
| **Mitotic mutants Subclass 2:** block to nuclear division in mitosis | 11C9 | 0-1 divisions/ 1 & 2 parasites per vacuole | <10-6 | 1) large parasite size but normal shape.  2) nucleus:parasite ratios=1:1.  3) arrest of internal daughters.  4) TEM micrographs show defective early daughter formation. | 1) predominant 2N-2.3N DNA content. |
|  |  |  |  |  |  |
| **Budding defects: Subclass 1:**  early bud arrest | 118G4 | 0-1 divisions/ 1 & 2 parasites per vacuole | <10 -6 | 1) large parasite size but normal shape.  2) nucleus:parasite ratios=1:1.  3) small IMC1, but intense staining bodies consistent with early bud arrest.  4) PCNA stain consistent with G1 nucleus with <5% S phase. | 1) predominant 1N DNA content with <5% 1.8N subpopulation. |
| most mutants in this subclass have predominant 1N DNA contents which is consistent with premature budding | 57A1 | 0-1 divisions/ 1 & 2 parasites per vacuole | <10 -6 | 1) normal parasite size and shape.  2) nucleus:parasite ratios=major 1:1 & rare 2:1.  3) small IMC1 staining structures associated with mother outer membrane.  4) variable PCNA1 nuclear staining. | 1) predominant 1N DNA content with <5% 2N subpopulation. |
|  | 8 E3 | 0-1 divisions/ 1 & 2 parasites per vacuole | <10 -6 | 1) normal parasite size and shape.  2) nucleus:parasite ratios=1:1 & 2:1  3) small IMC1 staining structures-no late daughter buds.  4) PCNA1 nuclear staining variable. | 1) 80% 1N DNA content.  2) ~20% 2N DNA content. |
|  | 154G11 | 0-1 divisions/ 1 & 2 parasites per vacuole | <10 -6 | 1) normal parasite size and shape.  2) nucleus:parasite ratios= major 1:1, minor 2:1.  3) small dense IMC1 staining bodies.  4) variable PCNA1 nuclear staining. | 1) predominant 1N DNA content.  2) minor <1N and 2N subpopulations. |
|  | 27D12 | 0-1 divisions/ 1 & 2 parasites per vacuole | <10-5 | 1) parasites range from small to large but normal shape.  2) nucleus:parasite ratios=1:1, 2:1.  3) 20% parasites have two small IMC bodies at 4) variable nuclear PCNA1 staining. | 1) predominant 1N DNA content .  2) <5% 2N subpopulation. |
|  | 71G6 | 0-1 divisions/ 1 & 2 parasites per vacuole | <10-7 | 1) normal parasite size and shape.  2) nucleus:parasite ratio=1:1.  3) small, multiple IMC1 staining structures.  4) variable nuclear PCNA1 staining | 1) >95% parasites arrest with 1N DNA content. |
|  | | | | | |
| **Budding Defects Subclass 2:** late budding defects | 137D5 | variable divisions/ odd numbers of parasite per vacuole | <10 -6 | 1) irregular parasite size and shape.  2) nuclear division is unequal leading to some chromosome loss.  3) mother cell divides and retains the nucleus. Note that some plastids are not segregated into daughter buds and are lost into the vacuole.  4) nuclear PCNA1 staining variable. | 1) DNA contents at growth arrest ranges from 1N to 2N |
|  | 7A11 | variable divisions/ odd numbers of parasite per vacuole | <10-7 | 1) irregular parasite shape and size.  2) dapi staining indicates that nuclear division into daughter buds is defective.  3) in many instances daughter parasites are unable to resolve from the mother cell. | 1) variable DNA contents with predominant 1N, however, due to the instability of the aberrant budding forms these FACS data are an incomplete sample of the population. |
|  | | | | | |
| **Budding defects: Subclass 3:** uncoupling phenotype, multiple daughters | 42D6 | variable divisions/ odd numbers of parasite per vacuole | <10-7 | 1) large parasite size and irregular shape.  2) nuclear divisions are unequal, ratio of nuclei to daughters is variable and uncoupled.  3) abnormal daughters.  4) variable nuclear PCNA1 staining staining | 1) DNA content ranges from sub-1N to >1.8N FACS data are an incomplete sample due to instability of this mutant |
|  | 20C2 | variable divisions/ odd numbers of parasite per vacuole | <10 -6 | 1) irregular parasite shape and size.  2) nuclear divisions are unequal.  3) stoichiometry of nucleus to daughter buds is highly variable. | 1) DNA content variable from 1N to >2N. |
|  | 122C4 | variable divisions/ odd numbers of parasite per vacuole | <10 -6 | 1) irregular parasite size and shaped--catastrophic phenotype.  2) nuclear division is unequal with evidence of chromosome loss (formation of zoids) and chromosome re-replication leading to multiple nuclei.  3) daughter formation is defective in many parasites. | 1) DNA content of arrested parasites ranges from <1N to >2N.  FACS data are incomplete due to the instability of this mutant |
|  | PO-B3 | variable divisions/ odd numbers of parasite per vacuole | <10 -6 | 1) irregular parasite size and shaped. Parasites become progressively large over time.  2) nuclear division is unequal with different nuclei sizes.  3) centrosome duplication still occurs forgoing internal budding. | 1) three peaks present by FACS; 1N, 1.8-2N, and >2N. The ratio of 1N to 1.8-2N is slightly higher than a wild type. |
